# Left ventricular functional, structural and energetic effects of normal aging: Comparison with hypertension

**DOI:** 10.1371/journal.pone.0177404

**Published:** 2017-05-11

**Authors:** Jehill D. Parikh, Kieren G. Hollingsworth, Dorothy Wallace, Andrew M. Blamire, Guy A. MacGowan

**Affiliations:** 1 Institute of Cellular Medicine, Newcastle University, Newcastle upon Tyne, United Kingdom; 2 Newcastle Magnetic Resonance Centre, Newcastle University, Newcastle upon Tyne, United Kingdom; 3 Centre for In Vivo Imaging, Newcastle University, Newcastle upon Tyne, United Kingdom; 4 Institute of Genetic Medicine, Newcastle University, Newcastle upon Tyne, United Kingdom; 5 Department of Cardiology Freeman Hospital, Newcastle upon Tyne, United Kingdom; Universita degli Studi di Napoli Federico II, ITALY

## Abstract

**Objectives:**

Both aging and hypertension are significant risk factors for heart failure in the elderly. The purpose of this study was to determine how aging, with and without hypertension, affects left ventricular function.

**Methods:**

Cross-sectional study of magnetic resonance imaging and ^31^P spectroscopy-based measurements of left ventricular structure, global function, strains, pulse wave velocity, high energy phosphate metabolism in 48 normal subjects and 40 treated hypertensive patients (though no other cardiovascular disease or diabetes) stratified into 3 age deciles from 50–79 years.

**Results:**

Normal aging was associated with significant increases in systolic blood pressure, vascular stiffness, torsion, and impaired diastolic function (all P<0.05). Age-matched hypertension exacerbated the effects of aging on systolic pressure, and diastolic function. Hypertension alone, and not aging, was associated with increased left ventricular mass index, reduced energetic reserve, reduced longitudinal shortening and increased endocardial circumferential shortening (all P<0.05). Multiple linear regression analysis showed that these unique hypertensive features were significantly related to systolic blood pressure (P<0.05).

**Conclusions:**

1) Hypertension adds to the age-related changes in systolic blood pressure and diastolic function; 2) hypertension is uniquely associated with changes in several aspects of left ventricular structure, function, systolic strains, and energetics; and 3) these uniquely hypertensive-associated parameters are related to the level of systolic blood pressure and so are potentially modifiable.

## Introduction

In normal aging there are several well described changes in cardiovascular function. Vascular stiffness increases from young adulthood [1]. In the left ventricle diastolic function becomes impaired from middle age onwards, followed by changes in high energy phosphate metabolism, altered torsional strain patterns [2] and ultimately reduced stroke volume [3]. Heart failure is predominantly a disease of the elderly [4]. In approximately 30% of cases of those patients with heart failure admitted to hospital in the United Kingdom do not have left ventricular systolic dysfunction on echocardiogram [4], and so will often be diagnosed with heart failure with preserved ejection fraction (HF pEF). HF pEF shares several features of the normal aging responses in left ventricular function, illustrating how aging and cardiovascular diseases and their risk factors are closely linked [5].

How this accumulation of risk factors leads to HF pEF is not fully understood. A history of hypertension is a particular risk factor for HF pEF [6]. When comparing subjects with HF pEF and hypertensive heart diseases [7] it has been shown that there are similar levels of vascular and diastolic function abnormalities between these 2 groups, though what did distinguish them was the greater extent of left ventricular hypertrophy, and left atrial enlargement and dysfunction in the HF pEF group.

Whether hypertension increases the normal aging effects on cardiovascular function, and/or has other effects distinct from the normal aging process are unclear. In the current study we sought to address the hypothesis that hypertension leads to both exaggerated effects of aging on the left ventricle and also effects unique to hypertension, in terms of structure, function, high energy phosphate metabolism, and vascular stiffness. With that in mind, normal controls and hypertensive subjects (without other cardiovascular diseases or diabetes) were recruited in 3 age brackets by decades from the sixth to eighth decades.

## Materials and methods

### Subjects

Forty eight normal subjects were recruited into 3 discrete age bands, with 16 subjects in each decade of 50–59, 60–69 and 70–79 years (data from these subjects have in part been published previously, reference [8]). Forty subjects with hypertension were also recruited into these age categories with 15 between 50–59 years, 15 in 60–69 years and 10 in 70–79 years. Subject details are presented in Table 1. Normal subjects were defined as those without any cardiovascular diagnosis, diabetes mellitus or dialysis dependent renal failure. Normal subjects had a systolic blood pressure ≤150 mmHg and/or diastolic blood pressure ≤90 mmHg at a screening visit. Hypertensive subjects were defined as having a diagnosis of hypertension at a local general practice, though had no other cardiovascular diagnosis, diabetes or dialysis dependent renal failure. The subjects were screened with a 12-lead electrocardiogram, fasting lipid profile, and blood pressure measurements. All hypertensive patients were on anti-hypertensives, which were prescribed from the local General Practioner (GP). There were no significant differences in the number of anti-hypertensive agents between the 3 hypertension age groups (Table 1). The duration of hypertension treatment was similar across age groups. The combined use of angiotension converting enzyme inhibitors and angiotension receptor blocker medication was similar across age groups, though there was a higher usage of thiazide diuretics in older subjects (7% age 50–59 and 36% age 70–79). There were significantly higher levels of triglycerides, and lower levels of HDL and LDL levels in the hypertensive patients. 60% of the hypertensive patients were treated with a statin. Informed written consent was obtained for all patients, and this study was approved by a UK National Health Service Research Ethics Committee (NRES Committee North East—Newcastle & North Tyneside 1, reference number 12/NE/0057, and ClinicalTrials.Gov identifier NCT01504828). Subjects were recruited through a local Newcastle GP practice database and were studied between October 2013 and November 2015.

**Table 1:** Normal and hypertension subject details.

	Normals			Hypertension		
**Age Group**	50–59	60–69	70–79	50–59	60–69	70–79
**N**	16	16	16	15	15	10
**Gender** **(Female/Male)**	10/6	7/9	9/7	5/10	4/11	3/7
**Height (cm)**	172±9	170±10	164±10	172±10	169±8	167±10
**Weight (kg)**††	76±16	74±19	69±14	90±12	86±18	81±17
**BSA (m**^**2**^**)**†	1.8±0.2	1.8±0.2	1.7±0.2	1.9±0.2	1.9±0.2	1.8±0.2
**Heart Rate (bpm)**	60±10	56±10	61±11	62±15	63±8	60±5
**Chol (mmol/L)**‡	4.8±0.7	4.8±0.6	5.3±1.1	4.8±1.2	4.6±1.0	4.3±1.2
**TG (mmol/L)**††	0.8±0.6^a^	1.0±0.5	1.1±0.4	1.6±0.7	1.5±0.8	1.3±0.6
**HDL (mmol/L)**†	1.8±0.6	1.6±0.5	1.6±0.4	1.3±0.3	1.4±0.5	1.6±0.3
**LDL (mmol/L)**†	2.7±0.8	2.8±0.5	3.2±0.9	2.8±1.1	2.5±0.8	2.2±1.0
**Duration of HPTN (years)**				8±6	9±8	9±7
**% Thiazide**				7	13	36
**% ACEi**				60	33	45
**% ARB**				20	33	36
**% Alpha**				7	7	0
**% CCB**				20	33	27
**%BB**				7	13	9
**% on 1 med**				80	66	54
**% on 2 med**				20	34	46

BSA: body surface area; Chol: cholesterol; TG: triglycerides; HDL: high density lipoproteins; LDL: low density lipoproteins; HPTN: hypertension; ACEi: angiotensin converting enzyme inhibitor; ARB: angiotensin receptor antagonist; alpha: alpha-blocker; CCB: calcium channel blocker; BB: beta-blocker.

† P<0.05 and

†† P<0.01 normals vs hypertension

‡ P = 0.06 normals vs hypertension

^a^ P<0.05 vs corresponding hypertensive age group.

### Cardiac cine imaging

A Philips Achieva 3T scanner and a 6 channel receiver array were employed to acquire cardiac magnetic resonance imaging (MRI) data. Details of cardiac cine imaging and our algorithm for contour selection and calculating LV mass and systolic and diastolic parameters have been previously published [9] (S1 Appendix). The following hemodynamic parameters were derived: effective arterial elastance, a measure of afterload (Ea = end-systolic pressure (= systolic blood pressure x 0.9) / stroke volume normalised to body surface area), and end-systolic elastance, a measure of left ventricular systolic performance (Ees = end-systolic pressure / end-systolic volume normalised to body surface area [10]. Ventricular-arterial coupling is derived by the ratio of Ea/Ees, which is a dimensionless number as both have the same units. Ventricular-arterial coupling measures the balance between properties of the left ventricle and the arterial circulation, and a decrease in this ratio may suggest that left ventricular properties are a more dominant abnormality compared to levels of afterload [10].

Assessment of diastolic function from cine images was performed by calculating the ratio of peak early and late left ventricular filling rates (E/A ratio), and the early filling percentage was calculated as the volume increase from end-systole to the midpoint divided by the stroke volume*100 (EFP) (S1 Appendix).

Longitudinal shortening was determined in the four-chamber view by determining the perpendicular distance from the plane of the mitral valve to the apex in systole and diastole. The myocardial wall thickness at systole and diastole was determined at the same level as the cardiac tagging, and radial thickening was calculated.

### Magnetic resonance phase contrast pulse wave velocity

Pulse wave velocity (PWV) is a marker of vascular stiffness and is an important predictor of cardiovascular events [11]. Phase contrast MRI flow data were acquired at two slice locations in the descending aorta approximately 10 cm apart, using a high temporal resolution sequence to measure pulse wave velocity that has been described in detail previously [12,13] (S1 Appendix).

### Cardiac tagging and regional strains

**Fig 1:**
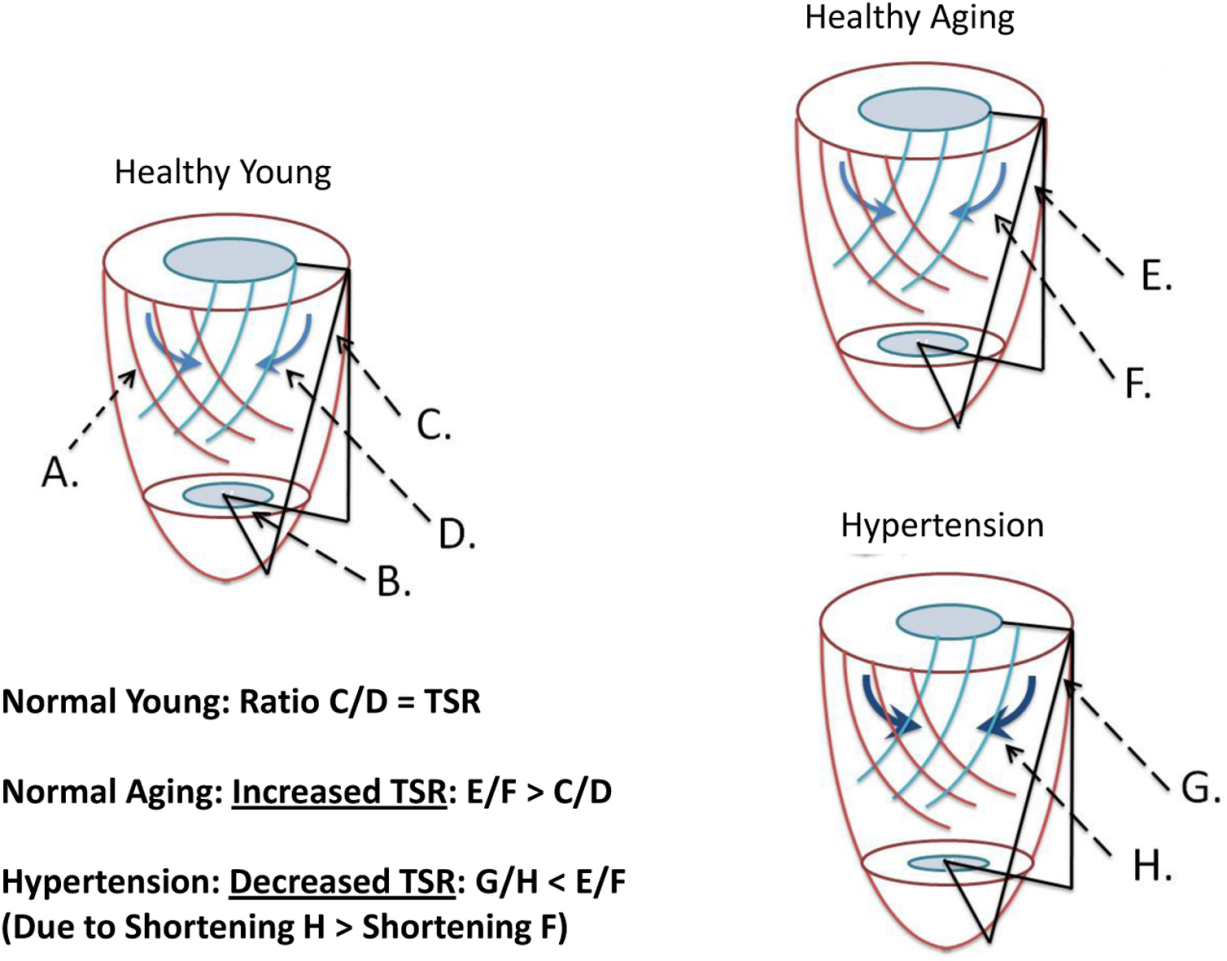
Healthy young: Illustration of epicardial torsion and endocardial circumferential shortening used in the calculation of the torsion to shortening ratio (TSR) and relationship to subepicardial and subendocardial fiber orientations. Epicardium is red and endocardium blue. **A.** Obliquely oriented subepicardial fibers produce rotation of the apex with respect to the base (**B.**) in a counterclockwise direction when looking from the apex to base, which is quantified in terms of the circumferential-longitudinal shear angle (**C.**). Epicardial torsion acts on the subendocardium with its greater mechanical advantage due to its larger radius, forcing subendocardial fiber bundles to shorten in a direction at almost 90° away from the subendocardial fiber direction (in the circumferential plane) (**D.**). This subepicardial to subendocardial interaction is quantified as the torsion to shortening ratio (TSR), and an increase in the TSR suggests reduced subepicardial influence over the subendocardium. **Healthy aging:** Rotational angle **E** and torsional angle **F** are increased compared to healthy young, there is no change in endocardial circumferential shortening and so the TSR is increased indicating reduced interactions between subepicardium and subendocardium. **Hypertension:** Rotational and torsional angles are unchanged compared to healthy young (angle **G**), though there is increased endocardial circumferential shortening (thick blue arrows, shortening **H**) indicating increased interaction between the subepicardium and subendocardium and therefore TSR is decreased.

Cardiac tagging works by applying radiofrequency pulses to cancel MR signal from the myocardium in diastole in a rectangular grid pattern and tracking the deformation of these tags through the rest of the cardiac cycle. Two tagged short axis images were obtained at the same session as previously described [2,14] (S1 Appendix). The Cardiac Image Modelling package (University of Auckland) was used to analyse the tagging data by aligning a mesh on the tags between the endo- and epicardial contours, and is described in detail elsewhere [9]. The epicardial torsion between the two short axis planes (taken as the circumferential-longitudinal shear angle defined on the epicardial surface) was calculated (Fig 1). Circumferential strain was measured at the epicardial, mid and endocardial thirds of the myocardium. The ratio of the peak torsion (in radians) and the peak circumferential strain in the endocardial third of the myocardium (%) was derived and is referred to as the torsion to shortening ratio, TSR (radians) [15,16]. The TSR is a measure of the ability of the subepicardium (as measured by torsion) to exert a mechanical advantage over the subendocardium (as measured by subendocardial circumferential shortening). In normal ageing this ratio increases, suggesting that there is systolic subendocardial dysfunction (Fig 1) [2,15].

### Cardiac spectroscopy

Cardiac high-energy phosphate metabolism (phosphocreatine to adenosine triphosphate, PCr/ATP ratios) was assessed using ^31^P magnetic resonance spectroscopy. The PCr/ATP ratio is a marker of energetic reserve, as phosphocreatine is the source of phosphorous for ATP. A decrease in this ratio has been associated with an imbalance between oxygen supply and demand in the myocardium [17] and adverse events in heart failure [18]. Data were collected using the same 3T Achieva scanner (Philips, Best, NL) with a 10cm diameter ^31^P surface coil (Pulseteq, UK) for transmission/reception of signal, and has been described in detail previously [2,19] (S1 Appendix).

### Data and statistical analysis

To compare the effects of hypertension and aging, 2-way analysis of variance was used with hypertension and age groups as the two factors (each factor being a categorical variable). Additional analysis also examined the effects of gender. No interaction effects were seen between the factors, so only hypertension and age effects are reported. Post hoc testing was performed with the Scheffé test. Multiple linear regression analysis was used to determine predictors of variables associated with hypertension. Differences in proportions were tested with the Chi Squared test. All statistics were performed using SPSS (version 22). Data are presented as mean ± standard deviation, and *P*<0.05 was considered statistically significant.

## Results

### Systolic blood pressure, vascular stiffness and ventricular-arterial coupling (Table 2)

There were significant increases in systolic blood pressure and Ees with age with additional effects of hypertension (Table 2). PWV and Ea increased with age but not with hypertension. Ea/Ees was reduced in the hypertension group, reflecting primarily as a consequence of the higher levels of Ees. This suggests that left ventricular systolic properties are the more dominant abnormality in this group of patients with treated hypertension compared to vascular abnormalities.

**Table 2:** Pressures, vascular stiffness measures, afterload, and diastolic function in normal and hypertensive patients by age group.

	Normals				Hypertension	
Age Group	50–59	60–69	70–79	50–59	60–69	70–79
**Pressure, Vascular Stiffness and VA Coupling**						
Systolic BP (mmHg)**/††	123±8	127±15^a^	135±11	139±10	144±11	151±12
**Diastolic BP (mmHg)**	69±10	67±7	72±9	73±10	72±6	72±7
PWV (m/s)*	6.1±1.6	7.3±2.4	8.3±1.7	6.5±2.1	7.9±2.8	7.5±4.0
Ea (mmHg/mL)**	2.6±0.7	2.8±0.9	3.3±1.2	2.8±0.8	3.2±0.5	3.8±1.3
Ees (mmHg/mL)*/††	6.2±2.7	6.2±2.7	7.8±2.8	7.3±2.0	8.1±2.1	9.9±2.7
**Ea/Ees** †	0.46±0.09	0.49±0.13	0.48±0.20	0.38±0.09	0.41±0.14	0.39±0.13
**Diastolic Function**						
E/A**	1.9±0.9	1.5±0.7	0.9±0.2	1.8±0.8	1.4±0.4	1.3±0.8
EFP (%)**/††	72±10	69±6 ^a^	61±7	68±8	58±6	60±11

BP: blood pressure; PWV: pulse wave velocity, Ea: arterial elastance, Ees: end-systolic elastance; Ea/Ees: Ventriculo-arterial coupling; E/A ratio of early to late peak filling rates, EFP: early filling percentage.

* P<0.05 and

** P<0.01 for age effect

† P<0.05 and

†† P<0.01 normals vs hypertension

^a^ P<0.05 vs corresponding hypertensive age group.

### Diastolic function (Table 2)

Diastolic function, as determined by the E/A ratio and EFP, was impaired with aging (i.e., reduced values with aging). While hypertension did not have an additional (independent) effect on the E/A ratio, it further reduced EFP.

### Left ventricular structure, function, and energetics (Table 3)

Left ventricular mass index, and ejection fraction were increased and PCr/ATP reduced with hypertension only, without effects of aging. End-diastolic volume index and stroke volume index were borderline reduced with age without any additional effect of hypertension. There was a borderline reduction in end-systolic volume index in hypertension.

**Table 3:** Measures of left ventricular structure, systolic function, energetics and strains in normal and hypertensive patients by age group.

	Normals			Hypertension		
Age Group	50–59	60–69	70–79	50–59	60–69	70–79
**LV Structure, Global Systolic Function and Energetics**						
**LV Mass Index (g/m**^**2**^**)** ††	64±13	65±11	61±10	79±19	78±13	73±20
**End-Diast. Vol Index (mL/m**^**2**^**)**§	68±18	65±15	60±14	68.0±15	61±12	55±16
**End-Syst. Vol Index (mL/m**^**2**^**)**‡	23±8	23±8	20±8	20±6	19±8	16±5
**Stroke Vol Index (mL/m**^**2**^**)**§	45±12	42±9	40±9	48±11	41±7	39±12
**Ejection Fraction (%)**†	67±5	65±6	67±9	70±6	69±6	70±6
**PCr/ATP** ††	1.89±0.28	1.90±0.34	1.90±0.45	1.72±0.62	1.54±0.19	1.45±0.43
**Systolic Torsion and Strains**						
**Epi. Circ. Strain (%)** ††	12.0±3.5	13.1±2.9	11.6±1.9	11.6±2.9	9.5±2.5	9.8±2.5
**Endo. Circ. Strain (%)** ††	24.8±3.7	26.1±4.6	24.2±2.8	29.7±3.3	28.9±4.8	28.6±3.8
**Torsion (**^**o**^)*	5.7±1.0	7.9±2.1	7.7±2.7	6.5±1.4	7.2±1.4	6.7±2.6
TSR (radians)*/ ††	0.41±0.10	0.54±0.13	0.56±0.21	0.39±0.11	0.44±0.11	0.41±0.15
**Long. Shortening (%)** ††	20.0±3.7^a^	19.0±4.0	19.2±4.4	14.7±3.2	15.7±3.7	14.1±2.2
**Radial Thickening (%)**	59.0±16.1	66.8±17.9	71.0±16.0	64.1±31.1	57.3±13.4	56.8±14.3

LV: left ventricular; Diast: diastolic; Vol: volume; Syst: systolic; PCr/ATP: phosphocreatine to adenosine triphosphate ratio; epi: epicardial: mid: midwall; endo.: endocardial; circ.: circumferential; TSR: torsion to shortening ratio; long: longitudinal.

* P<0.05 and

** P<0.01 for age effect

† P<0.05 and

†† P<0.01 normals vs hypertension

‡ P = 0.05 normals vs hypertension

§ P = 0.06 age effect.

^a^ P<0.05 vs corresponding hypertensive age group.

### Strains and torsion (Table 3 and Fig 1)

With hypertension there was a significant redistribution of systolic strains. Epicardial circumferential shortening decreased, while endocardial circumferential shortening increased. Midwall circumferential shortening was not significantly changed by age or hypertension (S1 Dataset). Additionally, longitudinal shortening was reduced in the hypertension group. With aging, there was an increase in torsion. As endocardial circumferential shortening was unchanged with normal aging, the TSR was increased with aging, indicating reduced ability of the subepicardium to affect subendocardial shortening as previously described [2,15]. However, the opposite effect was seen in hypertension. As endocardial circumferential shortening was increased, and torsion unchanged, TSR decreased. This suggests enhanced subendocardial function with increased interactions between the subepicardium and subendocardium in hypertension. Radial thickening was not significantly different with either age or hypertension.

### Effects of gender

Females had significantly lower left ventricular mass index (62.3 ± 10.6 vs 77.4 ± 14.8 g/m^2^, P<0.01), and higher ejection fraction (66 ± 6 vs 69 ± 6, P<0.05). All circumferential strains (whole, epicardial, midwall and endocardial) were elevated in females (e.g., whole wall: 19.5 ± 2.3 vs 17.8 ± 3.5%, P<0.01).

### Predictors of hypertension-related effects on left ventricular mass index, longitudinal shortening, endocardial circumferential shortening and PCr/ATP ratio (Table 4)

**Fig 2:**
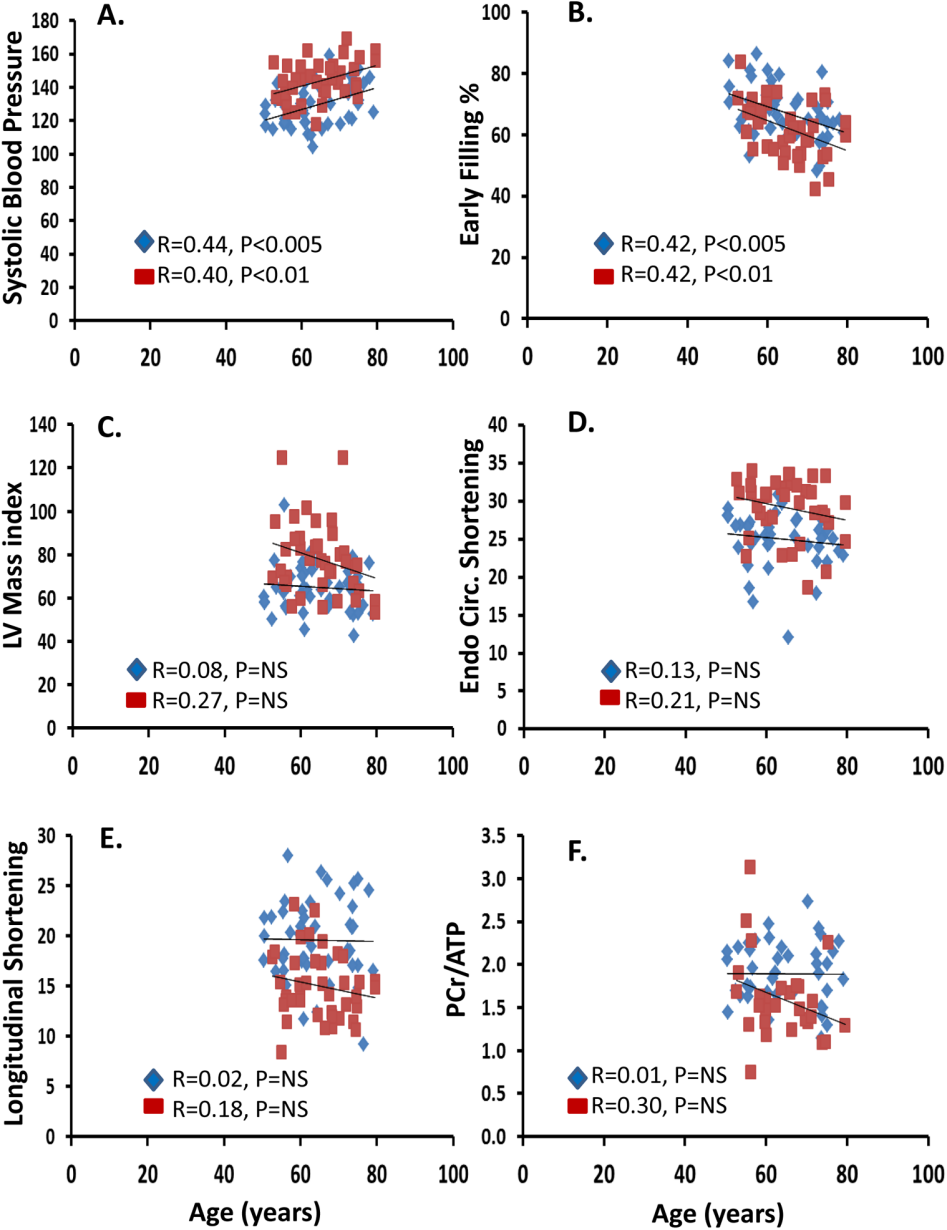
**Scatter plots of the effects of aging on A. systolic blood pressure, B. early filling percentage (measure of diastolic function), C. left ventricular mass index, D. endocardial circumferential (endo. circ.) shortening, E. longitudinal shortening and F. PCr/ATP ratio.** Both aging and hypertension are associated with significant effects on A and B (linear regression slope increases in both groups and shifted parallel in hypertension), however for C-F there is no significant relationship of aging. Normal subjects are in blue and hypertensive patients in red.

We concentrated further analysis on 4 parameters that were uniquely and highly significantly associated with hypertension: left ventricular mass index, longitudinal shortening, endocardial circumferential shortening, and the PCr/ATP ratio. Fig 2 illustrates that there is a significant effect of age on systolic blood pressure and diastolic function (early filling percentage), and that this effect is added to by the diagnosis of hypertension (Fig 2A and 2B with parallel shift in linear regression line that is also significantly related to age). However, there is no relationship of aging to left ventricular mass index, longitudinal shortening, endocardial circumferential shortening, or the PCr/ATP ratio (Fig 2C–2F), showing that these parameters are uniquely related to hypertension only (as shown with ANOVA statistics in Tables 2 and 3).

**Table 4:** Multiple linear regression analysis of four principal factors associated with hypertension: left ventricular mass index, longitudinal shortening, endocardial circumferential shortening, and the PCr/ATP ratio.

Dependent Variable:	LV Mass Index:		Long. Short.		Endo. Circ. Short.		PCr/ATP	
	beta	P =	beta	P =	beta	P =	beta	P =
**Age**	-0.141	0.189	0.144	0.293	-0.107	0.448	0.031	0.862
**Gender**	-0.516	**0.000**	0.089	0.537	0.265	0.086	-0.336	**0.065**
**BSA**	0.090	0.416	-0.177	0.221	0.202	0.185	-0.159	0.398
**Heart Rate**	0.153	0.117	-0.109	0.372	-0.041	0.747	0.219	0.166
**Systolic P**	0.391	**0.001**	-0.443	**0.004**	0.335	**0.031**	-0.590	**0.001**
**Diastolic P**	-0.185	**0.068**	0.092	0.503	-0.272	**0.049**	0.307	**0.048**
**VA Coupling**	0.242	**0.006**	0.021	0.848	-0.321	**0.007**	0.050	0.705
**PWV**	0.088	0.294	-0.063	0.569	0.050	0.669	-0.027	0.837
**EFP**	-0.194	0.086	0.136	0.323	0.035	0.811	-0.271	0.126
**SVi**	0.621	**0.000**	**-**	-	-	**-**	0.397	**0.029**
	**(R**^**2**^ **=** 0.545)		**(R**^**2**^ **=** 0.184)		**(R**^**2**^ **=** 0.190)		**(R**^**2**^ **=** 0.164)	

LV: left ventricular; long: longitudinal, short: shortening; endo: endocardial; circ: circumferential; PCr/ATP: phosphocreatine to adenosine triphosphate ratio; BSA: body surface area; P: pressure; VA: ventricular-arterial; PWV: pulse wave velocity; EFP: early filling percentage; SVi: stroke volume index.

We used multiple linear regression analysis from the whole dataset combining normal and hypertensive patients to determine predictors and potential mechanisms of these 4 principal effects of hypertension. Variables included in the model were basic demographic factors: age, gender, BSA; vascular: systolic and diastolic blood pressure, Ea/Ees and pulse wave velocity; diastolic function: early filling percentage; systolic function: stroke volume index; and heart rate. Stroke volume index was not included in the models with longitudinal and endocardial circumferential shortening as these are all direct manifestations of systolic function. Likewise, we did not perform a multiple linear regression analysis for ejection fraction as it is closely related to stroke volume and other systolic strains.

For left ventricular mass index as dependent variable, significant predictors were gender (lower mass in females), systolic blood pressure (positive relationship), Ea/Ees (positive), and stroke volume index (positive). This suggests that higher levels of left ventricular mass in this mixed group of normals and hypertensives are associated with higher systolic blood pressure and adverse ventricular-arterial coupling, but increased left ventricular mass preserves stroke volume. This model accounted for over 50% of the variance associated with left ventricular mass index.

**Fig 3:**
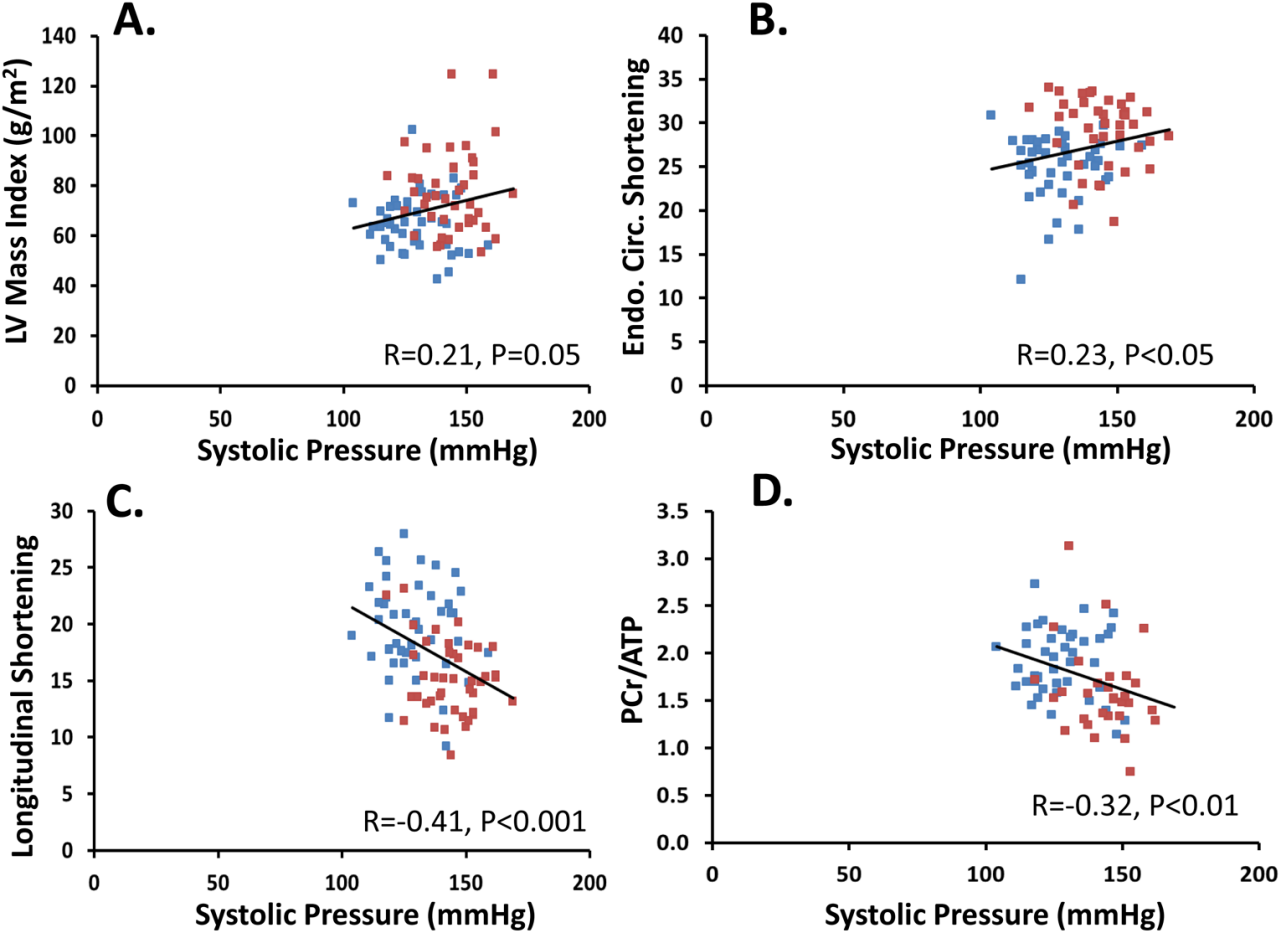
**Scatter plots of A. left ventricular (LV) mass index, B. endocardial circumferential (endo. circ.) shortening, C. longitudinal shortening and D. PCr/ATP ratio versus systolic pressure with univariate linear regression analysis.** Normal subjects are in blue and hypertensive patients in red. The regression line refers to the combined cohort.

For the other 3 models, there were also significant relationships with systolic blood pressure for all the dependent variables. For longitudinal shortening, the opposite effect of systolic blood pressure was seen compared to left ventricular mass index, in that longitudinal shortening decreased as systolic blood pressure increased. With endocardial circumferential shortening, higher levels of systolic blood pressure were associated with higher values of endocardial circumferential shortening. Higher levels of systolic blood pressure were also significantly associated with lower levels of PCr/ATP. Fig 3 illustrates scatter plots of these 4 dependent variables versus systolic pressure with univariate linear regression analysis.

## Discussion

**Fig 4:**
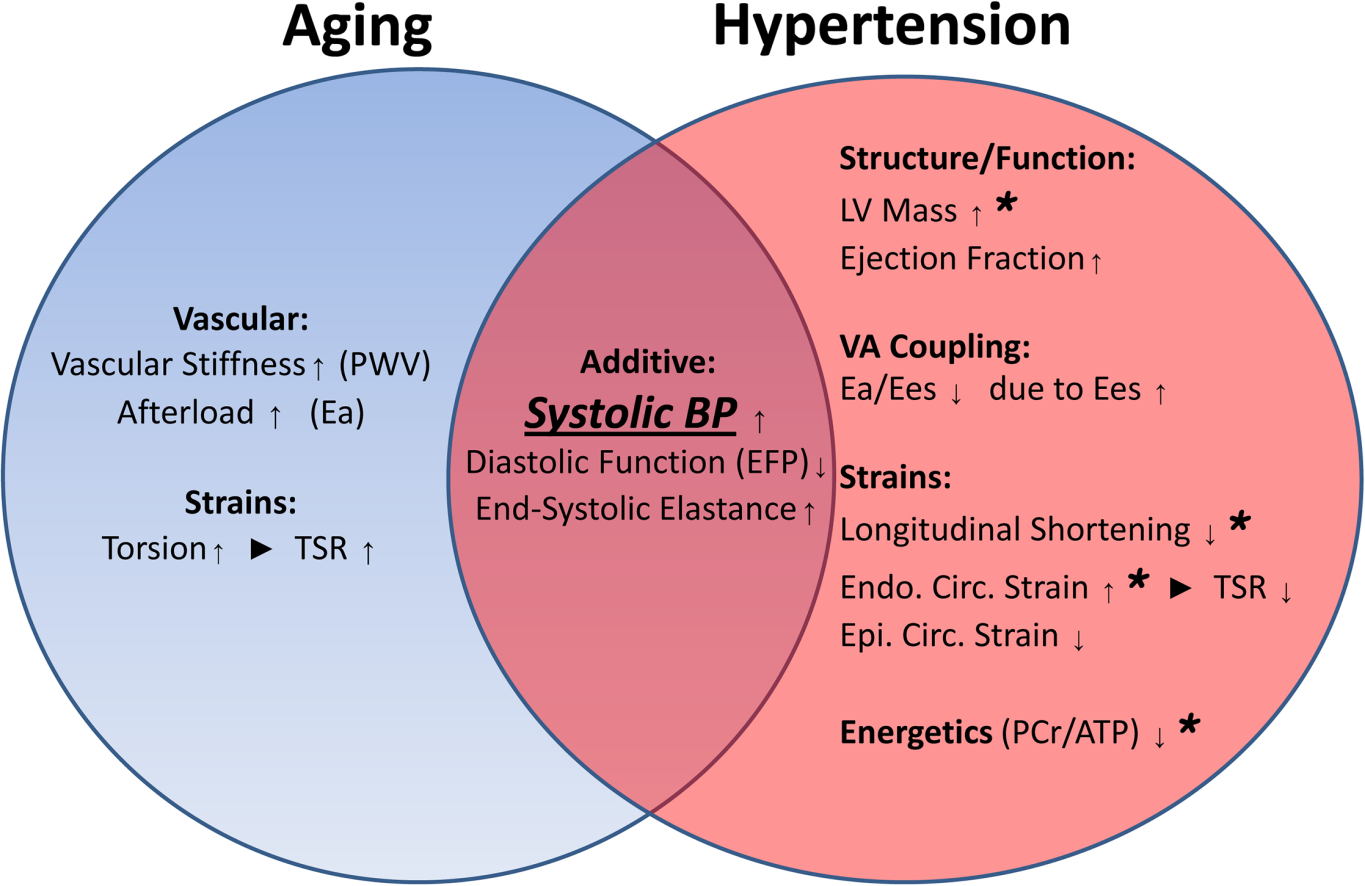
Summary results figure. Both aging and hypertension are associated with unique effects, and additive effects are shown in the overlapping center. In particular, systolic blood pressure has a significant relationship to left ventricular mass index, longitudinal shortening, endocardial circumferential shortening, and the PCr/ATP ratio (highlighted with ‘*’) suggesting that these effects are potentially modifiable. Abbreviations as in Tables 2 and 3.

In this study we demonstrate in a group of normal subjects and patients with treated hypertension (though no other cardiovascular diagnosis or diabetes) that hypertension has both additive effects to the normal effects of the aging process, and also effects uniquely associated with hypertension (Fig 4). Increases in systolic blood pressures and Ees seen with aging are added to by hypertension, and diastolic function is also further impaired (EFP). Unique effects associated with hypertension are increases in left ventricular mass index, increase in ejection fraction, reduction in longitudinal shortening, redistribution of circumferential strains with reduced epicardial and increased endocardial shortening, and impaired energetics (PCr/ATP). Unique effects of aging are increases in PWV and Ea, reduction in the E/A ratio, and increase in torsion. The TSR is a measure of the subepicardial influence over the subendocardium, with reduced subepicardial effects on subendocardial shortening increasing TSR (Fig 1). There are opposite effects with aging and hypertension. In aging this ratio is increased. In hypertension, this ratio is decreased due to enhanced ability of the subepicardium to effect subendocardial shortening. The reduction in the ratio of Ea/Ees (VA coupling) in hypertension, due to the increase in Ees highlights that the dominant abnormalities that we see in hypertension are left ventricular as opposed to afterload or vascular stiffness which were not significantly different between these treated hypertension subjects and aged-matched controls.

### Mechanisms of hypertension effects: Role of systolic blood pressure and stroke volume

Systolic blood pressure appears particularly important in the multiple linear regression analysis. Higher levels of systolic blood pressure are related to increased left ventricular mass, reduced longitudinal and endocardial circumferential shortening and reduced PCr/ATP. Also higher levels of stroke volume index are strongly related to higher levels of left ventricular mass index. This suggests that increases in left ventricular mass help preserve stroke volume in the presence of higher systolic pressure. The hypertensive patients were on treatment though these data suggest that despite treatment, there was potential to reduce effects of hypertension on the left ventricle by more intensive reduction of systolic blood pressure. Recently the Systolic Blood Pressure Intervention Trial (SPRINT) [20] has shown that intensive lowering of blood pressure reduces a composite end-point of several cardiovascular outcomes, and as a secondary outcome there was a significant reduction in heart failure. Our data suggest that more intensive lowering of blood pressure could potentially reduce adverse effects of hypertension by reducing left ventricular mass and endocardial circumferential shortening, and improving cardiac energetics and longitudinal function. Increases in left ventricular mass are associated with adverse prognosis [21] and increased risk of heart failure [18]. Likewise reductions in PCr/ATP ratios in heart failure predict prognosis [22], and reduced longitudinal shortening also is an adverse prognostic marker in hypertension [23]. It should be noted that the target blood pressure in the standard treatment arm of the SPRINT trial was between 135–139 mm Hg, which is lower than a significant proportion of our hypertensive patients. To understand the mechanisms of the reduced heart failure with intensive blood pressure lowering as shown in the SPRINT trial, it would be important to study the effects of intensive blood pressure lowering with the imaging methods as used in this study.

### Altered systolic strains in hypertension

In the hypertensive patients we have documented reduced longitudinal shortening, increased endocardial circumferential shortening, preserved midwall circumferential shortening, and reduced epicardial circumferential shortening. Several conclusions can be drawn from these findings based on the anatomical fiber orientations in the left ventricle. Fibers are oriented at 74±3° at the subepicardium (with respect to the circumferential plane), circumferentially at the midwall, and -70±4° at the subendocardium [24,25]. Thus, midwall circumferential shortening is along the plane of the fiber orientation in that plane and so reflects fiber shortening. This is unchanged in the hypertensive subjects, suggesting systolic function at a fiber level is preserved in these patients. At the subendocardium, maximal shortening is close to 90° from the actual fiber direction in that plane (cross fiber shortening) and this is due to compression of subendocardial fiber bundles by the contracting outer layers of the left ventricle, exerting a greater mechanical advantage due to the greater radius [24,25] (Fig 1). In the hypertensive subjects, endocardial circumferential shortening is increased. As it is known that fiber orientations in hypertensive left ventricular hypertrophy are not different than normal hearts [26], this suggests increased endocardial cross fibre shortening and thus increased interactions from the epicardium on the endocardium. Consistent with that the TSR is reduced. There is greater shortening in the endocardial circumferential direction with correspondingly less shortening in the longitudinal direction. Epicardial circumferential shortening is reduced. As for the subendocardium, there are interactions from other layers of the left ventricle on the subepicardium, albeit to a much lesser extent (epicardial cross fiber shortening) [24]. Epicardial cross fiber shortening will be in a plane close to the epicardial circumferential plane, so the reduction in epicardial circumferential shortening implies a reduction in epicardial cross fiber shortening. Thus, subepicardial to subendocardial interactions are increased, while in the opposite direction they are reduced.

Preserved left ventricular systolic function and reduced longitudinal shortening are consistent findings in hypertension [27–30]. There are, however, apparent inconsistencies with respect to circumferential shortening. Using MR tagging, Palmon et al [30] have shown that circumferential shortening was reduced in all layers of the left ventricle in subjects with hypertensive left ventricular hypertrophy, albeit with a higher left ventricular mass/body surface area than the current study (127 ± 37 g/m^2^). In contrast to this, Narayanan et al [28] have shown using echocardiography that absolute strains were not significantly different between normals and hypertension in patients with mild hypertension (though also in a group of patients with higher LV mass than our cohort; LV mass index 89 ± 21, g/m^2^). Ahmed and colleagues [27] have shown with MR tagging in resistant hypertension (mean of 4 ± 1 medications, though similar LV mass index to our cohort 64 ± 18 g/m^2^), that circumferential strains were reduced, though no details were provided of variations through the left ventricular wall. Our data showing a more complex pattern in a group with relatively mild hypertension and mild left ventricular hypertrophy suggests that there may be an evolution of changes in circumferential shortening as hypertensive heart disease progresses–from preserved fibre shortening and increased endocardial circumferential shortening in mild hypertension (as seen in this study) to reduced fibre shortening and reduced circumferential shortening in all layers of the left ventricle in advanced hypertensive left ventricular hypertrophy.

### Elevated end-systolic elastance in aging and hypertension

End-systolic elastance is elevated both by normal aging, and this effect augmented by hypertension. This has been previously recognised [2,31]. In the context of aging and hypertension the significance of this finding is that it reflects stiffness of the left ventricle in systole (a passive property), as opposed to an increase in contractility (an active property) [31]. We have previously shown in a mouse model of muscular dystrophy cardiomyopathy that steroid-induced increases in left ventricular fibrosis are related to increases in end-systolic elastance [32]. In HF pEF increases in end-systolic elastance are further increased, and this enhanced slope of the relationship of pressure to volume may explain in part the susceptibility to clinical heart failure of these patients to increased blood pressure [33].

### Limitations

Our aging data are from a relatively short time span, limited by the age ranges when we can find community patients with hypertension, and are also cross-sectional. We have previously shown that the PCr/ATP ratio is reduced with aging over a wider range of ages [2], though there was no aging effect seen in this study. In that study we studied a separate group of patients from the ages of 20 and 69, though in the current study patients range from 50 to 79. The wider age range in the previous study allowed the detection of a significant effect of aging on the PCr/ATP ratio, that we could not reproduce in the smaller age range in the current study. The smaller age range in this study was specifically chosen to allow us to recruit subjects with hypertension at matched age ranges, as it is more difficult to recruit younger hypertensive patients. Nevertheless, the values in the hypertension patients in the current study are lower than the values for normal aging in the previous study suggesting that hypertension is indeed associated with impaired cardiac energetics. Also, these data are cross-sectional and so represent a snap shot of representative subjects at different ages, rather than a longitudinal progression. The hypertension patients were all treated according by local GP practices, and so there is some variation in medications used across the age groups, particularly with thiazide diuretics being more frequently prescribed in the older age group. We do not know how this may have affected measures of left ventricular function. The duration of hypertension was similar across the age groups, but additional longitudinal studies are required to determine how onset of hypertension at a younger fixed age (i.e. 40 years) effects our measurements in the 6^th^, 7^th^ and 8^th^ decades.

PWV was not increased in our hypertensive patients. Using arterial tonometry, hypertension is a significant risk factor for increased PWV [34]. Our findings of no increase in markers of vascular stiffness may in part be explained by the relatively small number of patients, but also because our hypertensive patients were all treated, had relatively mild hypertension, and had no other risk factors for increased vascular stiffness such as diabetes, other cardiovascular diagnoses or renal failure. Some of our normal patients may have been suitable for treatment with antihypertensive agents according to NICE guidance [35], which may result in an underestimation of the differences between the 2 groups.

Relaxation of the left ventricle is intrinsically related to afterload [36], and we have recently shown that in normal aging the E/A ratio is significantly related to afterload (as measured by effective arterial elastance [8]. Thus, the measurements of impaired relaxation may in part relate to changes in afterload.

### Conclusions

Hypertension, when treated and without other cardiovascular diagnosis or diabetes, is associated with significant changes in left ventricular structure, function and energetics in addition to normal aging effects. These are, at least in part, related to the level of systolic blood pressure, and so are potentially modifiable. Recent studies have suggested that intensive blood pressure lowering can improve left ventricular systolic and diastolic function [37], though the effects of intensive blood pressure lowering on the parameters that we have identified have not been studied. Also, beneficial effects on left ventricular function are less in older subjects [38], so it is important that younger hypertensive patients are targeted before the age-related changes in left ventricular function compound the effects of hypertension that we have demonstrated. Future studies should build on these data to determine how the accumulation of risk factors such as hypertension, diabetes, and aging result in heart failure in the elderly.

## Supporting information

S1 Appendix Appendix. (DOCX)

S1 Dataset Dataset. (XLSX)
