# Supplementary material for: Left ventricular functional, structural and energetic effects of normal aging: Comparison with hypertension
Source: PLoS One. 2017 May 11;12(5):e0177404. doi: 10.1371/journal.pone.0177404 (PMC5426746; doi:10.1371/journal.pone.0177404)
Supplement: S1 Appendix — (DOCX) [file pone.0177404.s001.docx]

**Parikh et al:**

**Supplementary Methods:**

**Cardiac MR cine imaging:** A dedicated six channel cardiac coil (Philips) was used with the subjects in a supine position and ECG gating (Philips vectorcardiogram). A stack of balanced steady-state free precession images was obtained in the short axis view during breath holding covering the entire left ventricle [field of view (FOV) = 350 mm, repetition time (TR)/echo time (TE) = 3.7/1.9 ms, turbo factor 17, flip angle (FA) 40^o^, slice thickness 8 mm, 0 mm gap, 14 slices, 25 phases, resolution 1.37 mm, temporal duration approximately 40 ms per phase, dependent on heart rate]. Image analysis was performed using the cardiac analysis package of the ViewForum workstation (Philips). Manual tracing of the epicardial and endocardial borders was performed on the short axis slices at end-systole and end-diastole. Manual tracing of the epicardial and endocardial borders was performed on the short axis slices at end-systole and end-diastole. The contours were reviewed by viewing the cine data with the contours attached. The basal slice selected for analysis for end-diastole and for end-systole occurred when at least 50% of the blood volume was surrounded by the myocardium. The apical slice was defined as the last slice showing intracavity blood pool. Papillary muscles were included in calculations of mass and excluded from calculations of volume. The interventricular septum was included as part of the left ventricle. Left ventricular mass, ejection fraction, and end-systolic and end-diastolic volumes were calculated. Myocardial mass was determined by multiplying the tissue volume by 1.05 g/cm^3^ (specific density of myocardium).

**Assessment of diastolic function from cine MR images:** To examine possible diastolic dysfunction, blood pool volumes were calculated across all phases to look for the characteristic two phase expansion of the blood pool. The papillary muscles were included in this determination. The left ventricular volume measurements (25 per cardiac cycle) were plotted against time. The data were then smoothed by using a piecewise cubic spline algorithm and oversampled into 256 data points to create a volume-vs.-time curve. The rate of change of blood pool volume was determined by taking the first derivative of this curve over the entire cardiac cycle. End-systole and end-diastole were defined as the times of lowest and greatest volumes, respectively. The time point halfway between end-systole and end-diastole was defined as the diastolic midpoint.

The following 2 indices were derived [1,2]: 1) peak early filling rate (defined as the maximum value of the first derivative between end-systole and the diastolic midpoint) and peak late filling rate (i.e., the maximum value of the first derivative between the midpoint and end-diastole). From these 2 parameters the early-to-late ratio is derived (i.e., the peak early rate divided by the peak late rate); 2) the early filling percentage (i.e., the volume increase from end-systole to the midpoint divided by the stroke volume 100).

**Phase contrast MRI to measure pulse wave velocity:** Phase contrast MRI acquisitions were specifically designed to calculate transit time (delta T) from time-velocity curves at two slice locations in the aortic arch and descending aorta, approximately 10 cm apart [3]. The following high temporal resolution sequence was used (TR/TE/FA/number of excitations (NEX)/slice thickness = 5 ms/2.9ms/100/1/8 mm, SENSE factor 2, field of view 300 mm× 225 mm, reconstructed voxel size 1.17 mm^2^, velocity encoding=150 m/s, 44 phases, breath hold duration ~19 s) at both slice locations.

**Cardiac tagging:** A turbo field echo sequence with an acceleration factor of nine was used (TR/TE/FA/NEX = 4.9/3.1/10°/1, SENSE factor 2, FOV 350×350 mm, voxel size 1.37×1.37 mm, tag spacing of 7 mm). Two adjacent short-axis slices of 10 mm thickness were acquired at mid-ventricle with a 2-mm gap. To calculate torsion (circumferential-longitudinal shear angle) the following formula is used (see figure 1 for definition of Angles C and B): Angle C = tan^-1^{2.r.Sin(Angle B/2)/d} (d = distance between the 2 short axis slices, and r = radius). The distance d is calculated as midway through the two slices (which are 10 mm thick, therefore 5 mm) plus the interslice gap of 2 mm, so = 5 + 5 + 2 = 12 mm.

**Cardiac spectroscopy:** Cardiac high-energy phosphate metabolism was assessed using ^31^P-MRS [4]. Data were collected using a 10-cm-diameter ^31^P surface coil (Pulseteq, UK) for transmission/reception of signal. Subjects were placed in a prone position and moved into the magnet so their heart was at magnet isocenter. Localizing images were collected using the in-built body coil to confirm location of the heart. Shimming was performed using a cardiac-triggered, breath-held field map. A slice-selective, cardiac-gated one-dimensional chemical shift imaging sequence was used with a 7-cm slice-selective pulse applied foot to head to eliminate contamination from the liver, with spatial presaturation of lateral skeletal muscle to avoid spectral contamination. Sixteen coronal phase-encoding steps were used, yielding spectra from 10-mm slices (TR = heart rate, 192 averages at the center of k-space with cosine-squared acquisition weighting, approximately 20 min acquisition time). Spectral locations were overlaid onto an anatomical image, and the first spectrum arising entirely beyond the chest wall was selected. Quantification of PCr, the gamma resonance of ATP, and 2,3-diphosphoglycerate (DPG) was performed using the AMARES time domain fit routine in the jMRUI processing software. After fitting, the ATP peak area was corrected for blood contamination by one-sixth of the amplitude of the combined 2,3-DPG peak, and the PCr/ATP ratios were calculated and corrected for saturation, with T1 relaxation time values of cardiac PCr and ATP taken from the literature. Flip angle correction was made using a gadolinium doped 20 mM phenyl phosphonic acid phantom at the center of the coil and a calibration dataset.

**Supplementary References:**

1. Kudelka AM, Turner DA, Liebson PR, Macioch JE, Wang JZ, Barron JT. Comparison of cine magnetic resonance imaging and Doppler echocardiography for evaluation of left ventricular diastolic dysfunction. *Am J Cardiol* 80: 384–386, 1997.
2. Mendoza DD, Codella NC, Wang Y, Prince MR, Sethi S, Manoushagian SJ, et al. Impact of diastolic dysfunction severity on global left ventricular volumetric filling - assessment by automated segmentation of routine cine cardiovascular magnetic resonance. J Cardiovasc Magn Reson. 2010;**12**:46.
3. Parikh JD, Hollingsworth KG, Kunadian V, Blamire A, MacGowan GA. Measurement of pulse wave velocity in normal ageing: comparison of Vicorder and magnetic resonance phase contrast imaging. *BMC Cardiovasc Disord*. 2016; **16:**50.
4. Hollingsworth KG, Blamire AM, Keavney BD, MacGowan GA. Left ventricular torsion, energetics, and diastolic function in normal human aging. *American Journal of Physiology-Heart and Circulatory Physiology* 2012; **302:**H885-H892.
